# Transmission of Asian Zika Lineage by *Aedes aegypti* and *Ae. albopictus* Mosquitoes in Florida

**DOI:** 10.3390/v15020425

**Published:** 2023-02-02

**Authors:** Rebecca A. Zimler, Barry W. Alto

**Affiliations:** Florida Medical Entomology Laboratory, Entomology and Nematology Department, Institute of Food and Agricultural Sciences, University of Florida, Gainesville, FL 32962, USA

**Keywords:** arbovirus emergence, invasive mosquitoes, Zika virus infection and transmission

## Abstract

The Asian lineage of Zika virus (ZIKV), a mosquito-borne pathogen originally from Africa, caused an epidemic into Brazil in 2015 and subsequently spread throughout the Americas. Local transmission in the U.S. is a public health concern, especially for Florida where the mosquito vectors *Aedes aegypti* and *Ae. albopictus* are widespread, abundant, and there is a high potential for virus introduction due to imported cases. Here we evaluate relative susceptibility to infection and transmission of Zika virus among geographic populations of *Ae. aegypti* and *Ae. albopictus* in Florida. Both species have been implicated as ZIKV vectors elsewhere, but both virus and vector genotype are known to influence transmission capacities and, hence, the risk of outbreaks. We test the hypothesis that *Ae. aegypti* and *Ae. albopictus* show geographic differences in midgut and salivary gland barriers that limit ZIKV transmission, using local populations of the two vector species recently colonized from three regions of Florida to compare their susceptibility to ZIKV infection, disseminated infection, and transmission potential. Susceptibility to infection was higher in *Ae. aegypti* (range 76–92%) than *Ae. albopictus* (range 47–54%). *Aedes aegypti* exhibited 33–44% higher susceptibility to infection than *Ae. albopictus*, with *Ae. aegypti* from Okeechobee, FL having 17% higher susceptibility to infection than *Ae. aegypti* from Miami, FL. Similarly, disseminated infection was higher in *Ae. aegypti* (range 87–89%) than *Ae. albopictus* (range 31–39%), although did not vary by region. Enhanced infection and disseminated infection in *Ae. aegypti* were associated with higher viral loads in mosquito samples than in *Ae. albopictus*. Transmission rates did not vary by species or region (range 26–47%). The results support the hypothesis that *Ae. aegypti*, but not *Ae. albopictus*, exhibited regional differences in midgut infection barriers. Our observation of higher vector competence for *Ae. aegypti* than *Ae. albopictus*, together with this species greater propensity to feed on humans, lends support to the notion that *Ae. aegypti* is regarded as the primary vector for ZIKV and public health concern in continental U.S.

## 1. Introduction

Native to Africa, the first signs of ZIKV emergence, in terms of increased human cases, began in Yap Island, Micronesia in 2007, followed by outbreaks in French Polynesia in 2013 [1] and an epidemic in northeastern Brazil associated with many humans infected, ranging from 440,000–1.3 million cases in 2015 [2]. During this time, ZIKV became a pandemic throughout the Americas, raising the concern about Zika transmission in the continental U.S., especially Gulf Coast states such as Florida [3].

A major epidemic of ZIKV in Florida would have terrible consequences for public health because of potential birth defects in babies and other neurological complications, such as Guillain-Barré syndrome. During 2015–2018, there were 1455 imported cases and 302 locally acquired Zika fever cases in Florida. Although travel associated Zika fever cases in Florida are sporadically identified, local transmission of ZIKV by mosquitoes has not been detected in recent years. Additionally, Florida is at a relatively higher risk than other states for local transmission of ZIKV because the putative vectors *Ae. aegypti* and *Ae. albopictus* [4,5] are abundant throughout much of the year. Most local transmission of ZIKV in the U.S. has occurred in the territories of Puerto Rico (91% of total), followed by American Samoa and the U.S. Virgin Islands [6]. Travel and commerce between U.S. territories where there is local transmission of ZIKV, and Florida is common and increases risks for imported Zika cases and a large-scale epidemic in the future. Furthermore, most cases of Zika fever are asymptomatic (80%) [6], which may further facilitate ZIKV transmission because the daily routine of the infected individuals will not be altered by illness. This study will provide essential and currently unknown information on the risk of ZIKV emergence in Florida and facilitate the development of a risk prediction map of Florida.

Zika virus (Family Flaviviridae, genus *Flavivirus*) is native to Africa and consists of three genetically distinct strains (lineages), one from Asia and two from Africa [7]. In December 2015 the first autochthonous transmission of ZIKV in South America was documented in northeastern Brazil. Phylogenetic analysis indicated that the ZIKV belonged to the Asian clade [8], which subsequently spread throughout the Americas [9]. Human infection with ZIKV causes high fever, rash, joint pain, conjunctivitis, headaches, and muscle aches, but complications may include serious life-threatening disease (Guillain-Barré syndrome and birth defects) [10,11]. Laboratory studies have shown that *Ae. aegypti* and *Ae. albopictus* are competent vectors of Zika virus [12,13,14,15,16,17]. In addition, *Ae. aegypti* has been found to be naturally infected with ZIKV in Malaysia, Senegal, Ivory Coast, and Brazil [5,18,19,20,21] and *Ae. albopictus* in Gabon [4], which implicates these species as probable ZIKV vectors to humans in the Americas.

A few studies, mostly from Africa, have demonstrated distinct differences in vector competence of *Ae. aegypti* and *Ae. albopictus* depending on ZIKV strain and geographic origin of the mosquitoes [22,23,24]. To date, only a few studies have assessed ZIKV infection and transmission in *Ae. aegypti* and *Ae. albopictus* from the Americas, including Florida [13,17]. An assessment of vector competence was made for *Ae. aegypti* from Brazil, French Guiana, Guadeloupe, Martinique, and Florida (Orlando) and for *Ae. albopictus* from Brazil and Florida (Vero Beach) at 28 °C. This study showed higher infection of *Ae. aegypti* (70–80%) than *Ae. albopictus* (20–85%) and variations in infection between the geographic populations were observed for *Ae. albopictus*, but not *Ae. aegypti*. Disseminated infection rates were much lower and varied by geographic population (*Ae. aegypti* 10–50%, *Ae. albopictus* 10–15%). Similarly, transmission rates were much lower (Brazilian *Ae. aegypti* 10%, Vero Beach *Ae. albopictus* 3%) than infection rates but were only tested for a subset of the geographic populations. Transmission rates for *Ae. aegypti* from Florida were not tested in this study [13]. While this study used F1-F2 generation *Aedes* from American populations, the Florida-derived *Ae. aegypti* and *Ae. albopictus* had been maintained as laboratory colonies for several generations (F7–10) and may not be representative of field populations. Vazeille et al. [25] showed that dengue-2 infection rates of *Ae. albopictus* were up to 4-fold lower in recently collected versus laboratory strains with more generations in the lab, suggesting that laboratory colonization altered susceptibility to infection. Laboratory colonization refers to the processes (acclimation and evolution) that likely occur during establishment of a caged population of mosquitoes for multiple generations. These former studies suggest huge variation in transmission potential among *Aedes* vector populations for ZIKV which is consistent with observations for transmission potential among vector populations for chikungunya virus [26]. Taken together, these observations suggest that midgut escape and salivary gland transmission barriers are the primary determinants of variation in vector competence among these *Aedes* vectors.

Few studies have evaluated vector competence of local populations of mosquitoes in Florida for ZIKV. Although *Ae. aegypti* has been implicated as the probable primary vector of ZIKV in the Americas [27], there are some circumstances in which *Ae. albopictus* may be the primary vector [4]. A study using *Ae. aegypti* and *Ae. albopictus* from Florida (F1-F2) and *Ae. aegypti* from the Dominican Republic identified differences in disseminated infection and transmission rates for two emergent lineages of chikungunya virus (CHIKV) belonging to the Asian and Indian Ocean lineages [28]. This study showed clear evidence of geographic differences in vector competence for *Ae. albopictus* between North, East, and West Florida (disseminated infection of Asian strain of CHIKV, range of 63–97%; transmission potential of Indian Ocean strain of CHIKV, range 12–71%) and for *Ae. aegypti* between South, East, West Florida and the Dominican Republic (disseminated infection of Indian Ocean strain of CHIKV, range of 64–91%; transmission potential of the Asian strain of CHIKV, range 18–63%). These results showed huge variation between the vector competence of these species depending on virus genotype as well as region-specific variation in infection and transmission across Florida. These results provide evidence that there may be variation in midgut and salivary gland barriers in *Ae. aegypti* and *Ae. albopictus* across Florida. Using freshly collected local populations from Florida, we test the hypothesis that *Ae. aegypti* and *Ae. albopictus* show regional differences in midgut and salivary gland barriers that limit ZIKV transmission [13].

## 2. Materials and Methods

### 2.1. Mosquitoes

**Figure 1:**
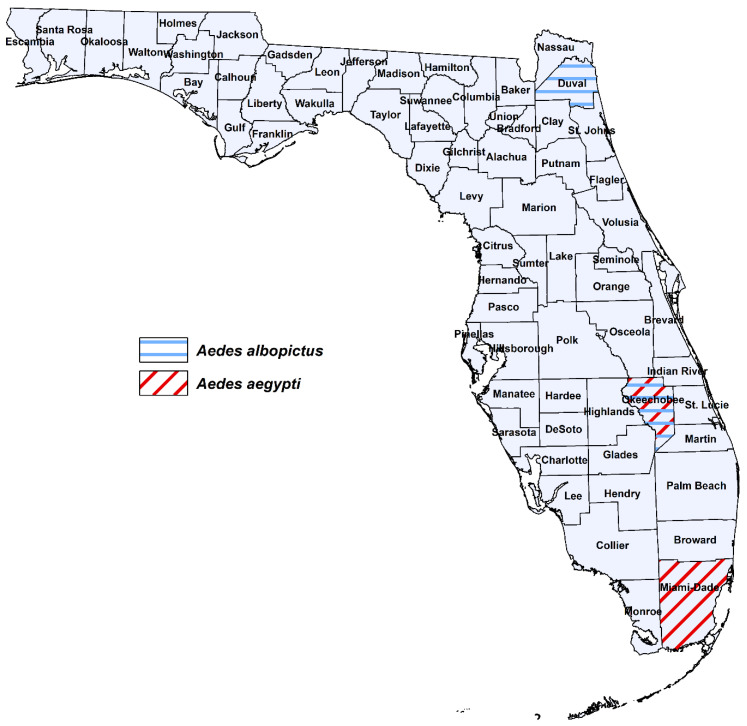
Geographic origin for Florida populations of *Ae. aegypti* and *Ae. albopictus* used in this study.

*Aedes aegypti* and *Ae. albopictus* were collected as larvae from cemeteries, tire shops or domiciliary settings across Florida where these species are present alone (allopatric) or coexist (sympatric) [29] (Figure 1, Table 1). We deliberately chose collection sites based on areas with past outbreaks of arboviruses (dengue) transmitted by these vector species as well as high risk areas for importation of ZIKV. Collection sites for *Ae. aegypti* included Miami-Dade (Miami) and Okeechobee (Okeechobee) Counties. Collection sites for *Ae. albopictus* included Duval (Jacksonville), and Okeechobee (Okeechobee) Counties. At the time of this study, *Ae. aegypti* and *Ae. albopictus* coexist in Okeechobee (sympatric) and exist alone (allopatric) in Miami (*Ae. aegypti*) and Duval (*Ae. albopictus*). We have identified variation in vector competence of these species across Florida for emergent strains of CHIKV, which may also exist for ZIKV [28,30]. 

Field-collected larvae were reared to adulthood on a diet of liver powder and lactalbumin at 28 °C. Pupae were transferred to vials with a cotton seal and upon eclosion identified to species. Female and male adults were provided with 10% sucrose solution, allowed to mate, and females ingested blood through membranes on commercially purchased bovine blood once per week in order to propagate eggs. The fourth-generation progeny of field-collected *Ae. aegypti* and *Ae. albopictus* were used for the ZIKV infection studies in the biosafety level-3 virology facility and Arthropod Containment level-3 at the Florida Medical Entomology Laboratory, in Vero Beach, FL. 

### 2.2. Virus Isolates and Propagation

A low passage strain of ZIKV from Puerto Rico (Asian lineage, GenBank: KU501215.1, strain PRVABC59) was used to infect the mosquitoes. An isolate of ZIKV was kindly provided to us by Centers for Disease Control and Prevention. The virus isolate was obtained in December 2015 from human serum. We deliberately chose this genotype of ZIKV because it is responsible for outbreaks in the Americas (starting in December 2015), and it is a risk for importation to Florida. Zika virus was propagated by inoculating monolayers of African green monkey (Vero) cells with 500 μL of diluted ZIKV from stock at a multiplicity of infection of 0.01 (number of viruses to cells). Viral inoculum was allowed to incubate for 1 h at 37 °C and 5% carbon dioxide atmosphere after which 25 mL media (Medium 199 supplemented with 10% fetal bovine serum, 0.2% penicillin/streptomycin, and 0.2% of the antifungal, Mycostatin) was added to each flask (T-175 cm^2^) with cells and incubated for an additional six days, after which ZIKV was combined with bovine blood and used in oral infection studies with mosquitoes.

### 2.3. Per Os Challenge of Mosquitoes

All oral challenge experiments used the same Zika virus genotype and 8- to 9-day old adult females were allowed to feed on ZIKV infected defibrinated bovine blood (Hemostat Laboratories, Dixon, CA, USA) mixed with adenosine triphosphate (ATP) at 0.005 M added as a phagostimulant to the infected blood meal. A Hemotek membrane feeding system (Discovery Workshop, Lancashire, UK) was used to allow mosquitoes to imbibe ZIKV infected blood [31]. We used a dose of ZIKV which is within the range of viremia levels experienced by infected humans (7log_10_ PFU/mL) [32,33]. Aliquots of blood were stored at −80 °C for later determination of virus titer in the oral challenge experiments. Zika virus was freshly propagated in monolayers of Vero cells in T-175 cm^2^ flasks for preparing the infected blood to feed to the mosquitoes (previous section). After the feeding trials, fully engorged females were held in cylindrical cages (10 cm height × 10 cm top diameter × 7 cm bottom diameter) along with an oviposition substrate and maintained under a 14 h: 10 h light: dark photo regime and 28 °C for 14 days. Adults were provided with 10% sucrose solution. The temperature chosen is representative of the average daily temperature observed in central Florida during late summer/early fall when arbovirus transmission is expected to occur [34].

To assess transmission potential and incidence of transmission, expectorated saliva was collected from mosquitoes. For this procedure, mosquitoes were individually transferred to 37-mL plastic tubes (h by d: 8 by 3 cm) 14 days post infection (dpi). Each tube held one mosquito and was fitted with a removable screen lid. No sucrose was provided to the mosquitoes for one day before the transmission trial. Each tube containing a mosquito was presented with a honey-soaked filter paper card fastened to the inside of the lid. The honey was dyed with blue food coloring which provided a visual marker. Mosquitoes were sorted based on the presence or absence of the blue marker in their crop, indicating that a mosquito fed on the honey and expectorated saliva during feeding. This system has been successfully used to measure transmission potential of ZIKV and CHIKV for *Ae. aegypti* and *Ae. albopictus* mosquitoes [17,28] as well as a field surveillance system to detect arboviruses that exploits the fact that female mosquitoes expectorate virus in their saliva during feeding on sugar sources [35]. Here we use this methodology as a proxy for potential to transmit ZIKV. Mosquitoes were examined using a flashlight for blue coloring in their crop after 24 h of the transmission assay. Mosquitoes and filter paper cards were collected 15 dpi and frozen at −80 °C for later analysis of expectorated saliva and virus using quantitative (q) RT-PCR methods [17]. Mosquitoes that did not feed on the blue honey were not tested for ZIKV transmission potential. Additionally, mosquitoes were individually dissected to separate the legs, and the bodies and legs were tested separately to determine the incidences of susceptibility to infection and disseminated infection through the presence of ZIKV RNA by quantitative RT-PCR used the CFX96 Real-Time PCR Detection System (Bio-Rad Laboratories, Hercules, CA, USA) and primers and probes specific to the Asian lineage of ZIKV. Mosquito samples were homogenized in centrifuge tubes with 1 mL of media and two steel BBs at 26 HZ for 3 min using a TissueLyser II (Qiagen, Germantown, MD, USA). Viral RNA was extracted using the QIAamp® Viral RNA Mini Kit from 140 µL samples (Qiagen, Germantown, MD, USA). The Superscript III One-Step qRT-PCR with Platinum® Taq Kit by Invitrogen (Invitrogen, Carlsbad, CA, USA), following the manufacturer’s protocol, was used to prepare RNA-extracted samples for quantitative real-time polymerase chain reaction (qRT-PCR) methods. Primers were designed to target the NS5 gene with the following sequences: forward primer, 5′-CTTCTTATCCACAGCCGTCTC-3′; reverse primer, 5′-CCAGGCTTCAACTCGTTAT-3′; and probe 5′-/56-FAM/AGAAGGAGACGAGATGCGGTACAGG/3BHQ_1/-3′ (Integrated DNA Technologies, Coralville, IA, USA). The program for qRT-PCR was as follows: 30 min at 50 °C, 2.0 min at 94 °C, 12 s at 94 °C, 1 min at 58 °C, and lastly repeated for 39 cycles. The cutoff for positive ZIKV samples was set at a Cq detection of 35 PCR cycles. Viral titer in mosquito samples were determined using a standard curve method that compares cDNA synthesis to a range of ZIKV serial dilutions in parallel with plaque assays of the same dilution of the virus, expressed as plaque forming unit equivalents (PFUE)/mL [36]. Testing for the presence of ZIKV RNA in bodies, legs, and saliva of mosquitoes enabled us to identify barriers to transmission (e.g., midgut infection and escape barriers, salivary gland barriers).

### 2.4. Statistical Analyses

Separate analyses were performed to test for treatment effects on susceptibility to infection (body samples), disseminated infection (leg samples), and transmission potential (saliva expectorates). Logistic regression analysis tested for species by region differences in infection parameters of mosquito samples (PROC LOGISTIC, version 9.4, SAS Institute Inc., Cary, NC, USA) based on the number of mosquitoes categorized for the presence or absence of ZIKV RNA. Viral loads in bodies, legs, and saliva were assessed for individual mosquitoes and analyzed by ANOVA (PROC GLM, version 9.4, SAS Institute Inc., Cary, NC, USA). Significant effects were followed by Tukey-Kramer multiple comparisons among treatment least-squares means for pairwise comparisons. Viral load in saliva expectorates is a proxy for amount of virus inoculated in a host during biting.

## 3. Results

### 3.1. Susceptibility to Infection

**Figure 2:**
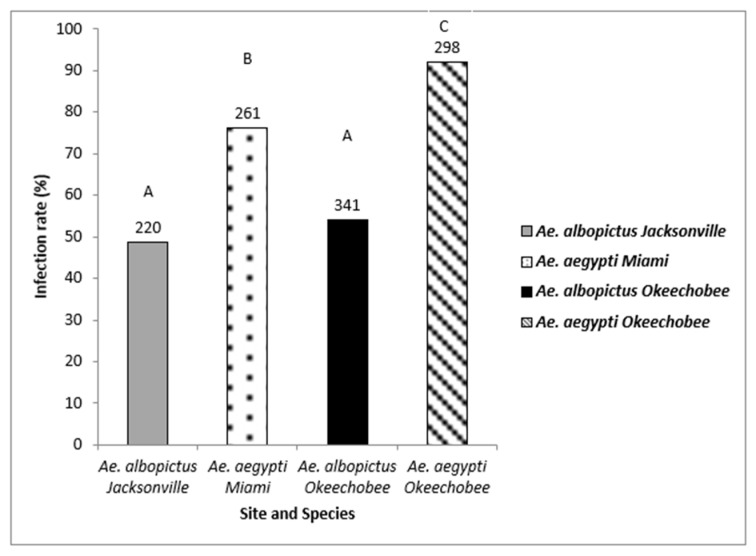
Zika virus infection (bodies) in *Ae. aegypti* and *Ae. albopictus* mosquitoes from different geographic populations in Florida were examined to determine susceptibility to Zika virus infection. The number of individual mosquitoes tested are shown above the bars. Comparisons of infection rates among treatment groups sharing the same letter are not significantly different from one another.

Oral challenge with Zika virus infected blood led to the establishment of infection in both *Ae. aegypti* and *Ae. albopictus*. Bodies were tested for the presence of ZIKV infection to determine the permissibility of infection (1120 mosquitoes: 559 *Ae. aegypti*, 561 *Ae. albopictus*) (Figure 2). Logistic regression analysis showed a significant effect indicating differences among the mosquito species and geographic populations (χ2 = 129.05, df = 3, *p* < 0.0001). Populations of *Ae. aegypti* (range, 75–90%) exhibited significantly higher rates of susceptibility to infection than populations of *Ae. albopictus* (range, 48–54%) (Figure 2, Table 2). Individuals of *Ae. aegypti* from Okeechobee were significantly more susceptible to ZIKV infection that *Ae. aegypti* from Miami (Figure 2, Table 2). The Jacksonville and Okeechobee populations of *Ae. albopictus* had similar infection rates to each other (range, 48–52%).

### 3.2. Disseminated Infection

**Figure 3:**
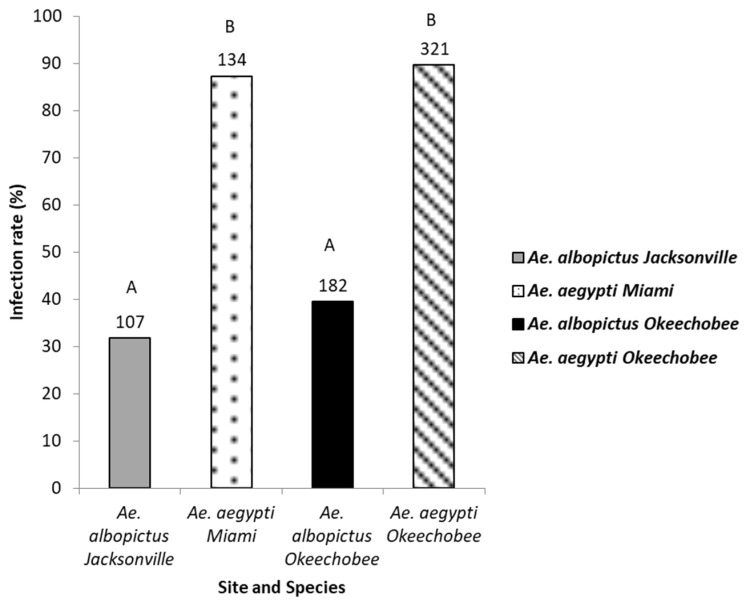
The legs of the Zika virus positive bodies of mosquitoes were tested for the presence of Zika virus. Leg infection in *Ae. aegypti* and *Ae. albopictus* mosquitoes from different geographic populations in Florida were examined to determine disseminated infection. The number of individual mosquitoes tested are shown above the bars. Comparisons of disseminated infection rates among treatment groups sharing the same letter are not significantly different from one another.

The legs of mosquitoes whose bodies were determined to be ZIKV positive were tested for disseminated infection (744 mosquitoes: 455 *Ae. aegypti*, 289 *Ae. albopictus*) to gauge differences in midgut escape barriers among treatment groups (Figure 3). Logistic regression analysis showed a significant effect indicating differences in disseminated infection among mosquito species and geographic populations (χ2 = 185.81, df = 3, *p* < 0.0001). Populations of *Ae. aegypti* (range, 88–90%) exhibited significantly higher rates of disseminated infection than populations of *Ae. albopictus* (range, 32–38%) (Figure 3, Table 2). Although the species exhibited distinct patterns of disseminated infection from each other, the populations within each species responded similarly. (Figure 3, Table 2).

### 3.3. Transmission Potential

**Figure 4:**
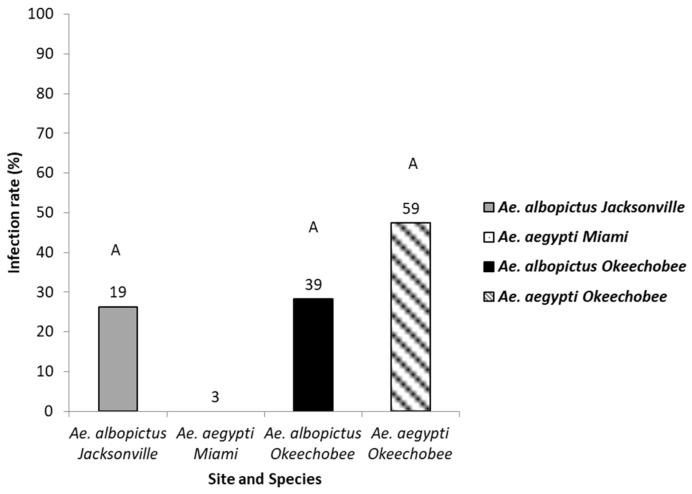
The saliva of mosquitoes with disseminated infection were tested as a proxy for transmission. Saliva infection in *Ae. aegypti* and *Ae. albopictus* mosquitoes from different geographic populations in Florida were examined to determine transmission potential. The number of individual mosquitoes tested are shown above the bars. Comparisons of saliva infection rates among treatment groups sharing the same letter are not significantly different from one another.

Detection of Zika virus in the saliva of *Ae. aegypti* and *Ae. albopictus* reinforced the belief that both these invasive mosquitoes are capable of transmitting Zika virus to humans. The saliva of mosquitoes that tested positive for disseminated infection and contained blue in the crop were tested for the presence of ZIKV (128 mosquitoes: 66 *Ae. aegypti*, 62 *Ae. albopictus*) (Figure 4). Logistic regression analysis showed no significant differences in saliva infection in *Ae. aegypti* (range, 0–48%) and *Ae. albopictus* (range, 28–29%) from the geographic populations (χ2 = 4.46, df = 3, *p =* 0.21, Table 2).

### 3.4. Zika Virus Titer in Mosquito Samples

**Figure 5:**
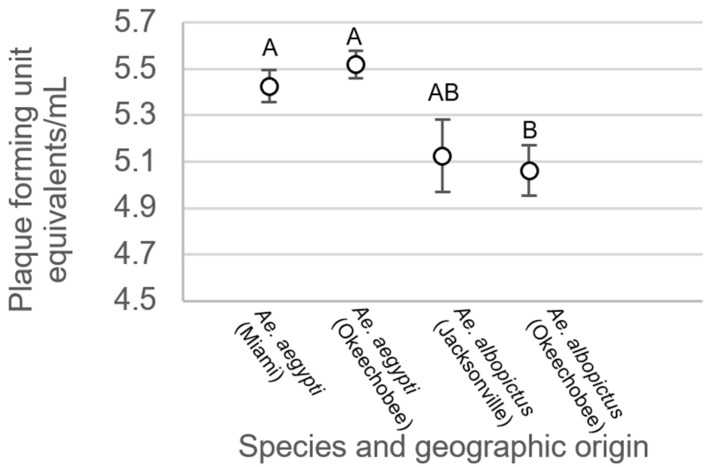
Zika virus titer in the bodies of *Ae. aegypti* and *Ae. albopictus* mosquitoes with disseminated infection from different geographic populations in Florida. Comparisons of viral titers among treatments sharing the same letter are not significantly different from one another.

ANOVA showed significant differences in body viral titer between treatment groups for mosquitoes exhibiting disseminated infection (F_3,543_ = 5.69, *p* > 0.0008). Overall, body titers were higher among *Ae. aegypti* than *Ae. albopictus* females. Specifically, body titer was significantly higher in mosquitoes of *Ae. aegypti* originating from Miami than individuals of *Ae. albopictus* originating from Okeechobee (Figure 5). Similarly, titer was significantly higher in individuals of *Ae. aegypti* originating from Okeechobee than individuals of *Ae. albopictus* from Okeechobee (Figure 5). All remaining pairwise comparisons were not significantly different from each other.

**Figure 6:**
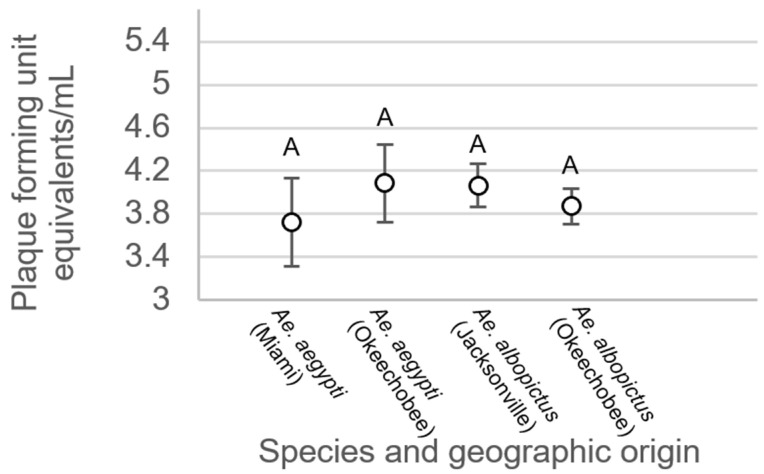
Zika virus titer in the bodies of *Ae. aegypti* and *Ae. albopictus* mosquitoes with non-disseminated infection from different geographic populations in Florida. Comparisons of viral titers among treatments sharing the same letter are not significantly different from one another.

**Figure 7:**
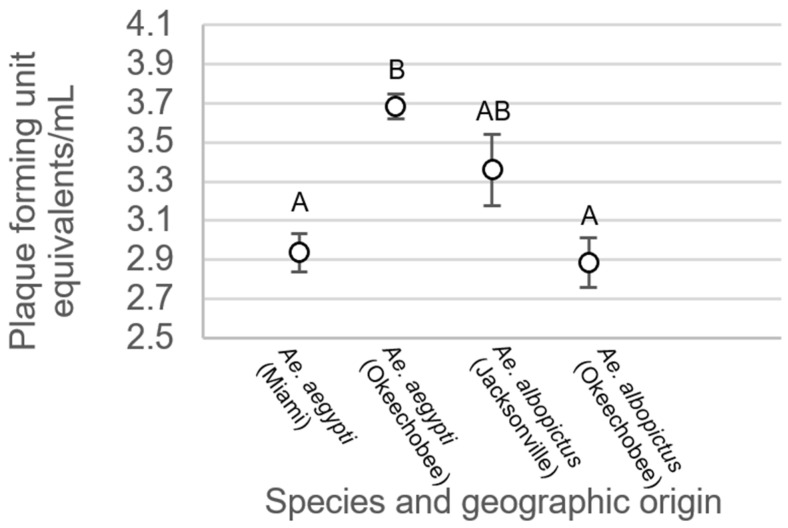
Zika virus titer in legs of *Ae. aegypti* and *Ae. albopictus* mosquitoes from different geographic populations in Florida. Comparisons of viral titers among treatments sharing the same letter are not significantly different from one another.

**Figure 8:**
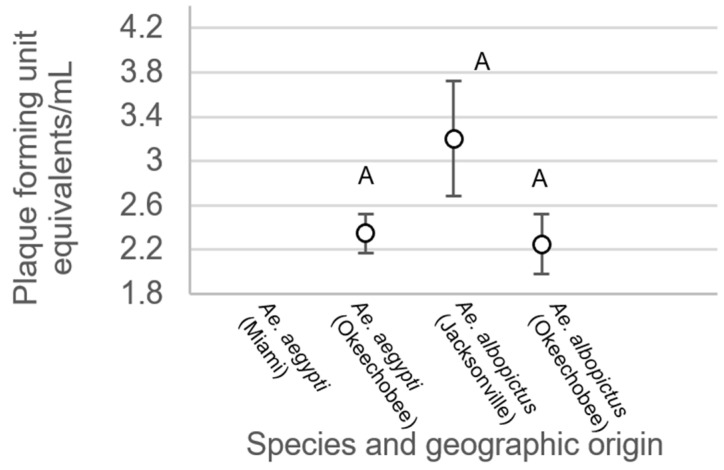
Zika virus titer in saliva of *Ae. aegypti* and *Ae. albopictus* mosquitoes from different geographic populations in Florida. Comparisons of viral titers among treatments sharing the same letter are not significantly different from one another.

For mosquitoes with non-disseminated infections, there were no significant differences between treatment groups (F_3,214_ = 0.34, *p* = 0.7997, Figure 6). Analysis of variance on viral titer in mosquito legs showed significant treatment differences (F_3,508_ = 19.48, *p* < 0.0001, Figure 7). Leg titer was significantly higher in individuals of *Ae. aegypti* from Okeechobee than titers of *Ae. aegypti* from Miami and *Ae. albopictus* from Okeechobee (Figure 7). No other significant differences were observed. Analysis of variance on saliva titer showed no significant differences between treatment groups (F_2,38_ = 1.38, *p* = 0.2629, Figure 8). However, too few samples were available to test for individuals of *Ae. aegypti* from Miami, which was left out of the analysis.

## 4. Discussion

The introduction of Zika virus from travel related cases continues to be reported in the U.S. Local transmission in the U.S. is a major public health risk, especially for Florida where mosquito vectors are abundant and there is a high potential for virus re-introduction. This study provides information on emergence potential of ZIKV in Florida and can help improve risk prediction for ZIKV in Florida by characterizing transmission efficiency of local populations of *Ae. aegypti* and *Ae. albopictus.* All geographic populations tested were susceptible to ZIKV and displayed disseminated infection, demonstrating the virus can pass through the midgut barrier(s) and progress to an advanced state of infection. We found limited support for our hypothesis of geographic differences in midgut and salivary gland barriers that limits Zika virus transmission. Specifically, support for the hypothesis was only observed for measurements of susceptibility to Zika virus infection in *Ae. aegypti*, and it did not change infectivity.

The observations from our study showed the Okeechobee population of *Ae. aegypti* as being highly susceptible to ZIKV infection. However, the same individuals from the Okeechobee population did not have enhanced disseminated infection and transmission rates compared to the other *Ae. aegypti* population, suggesting only a small enhancement of Zika transmission risk. However, the Okeechobee population of *Ae. aegypti* had similar or higher viral titers of legs and bodies of mosquitoes with disseminated infections than other groups of mosquitoes, perhaps attributable to enhanced viral replication. The current study did not assess the extrinsic incubation period of ZIKV, differences in behaviors (human biting rate), or other life history traits (adult survivorship) between geographic populations which limits our interpretation of anticipated changes to measures of risk of transmission (e.g., vectorial capacity), given that the observed difference being solely for susceptibility to ZIKV infection between two geographic populations of *Ae. aegypti*. However, we have identified variation in components of vector competence on a smaller spatial scale than previously observed for Zika [28]. However, we did not find any geographic population associated with higher risk for ZIKV transmission based on salivary infection. Lack of differences in saliva infection may be attributable to the low sample size and heterogeneity in the dataset, or that little variation exists for this phenotypic trait among these Florida mosquito populations. These observations suggest that midgut escape barriers may be playing an important role in the variation among these *Aedes* vectors. The relative higher vector competence for ZIKV by *Ae. aegypti*, and associated infectivity, can contribute to the likelihood of the two species transmitting ZIKV to humans in nature. 

Chouin-Carneiro et al. [13] found variation in susceptibility to ZIKV infection and disseminated infection in *Ae. aegypti* and *Ae. albopictus* from the continental United States and Brazil. Chouin-Carneiro et al. [13] observed higher infection and disseminated infection rates in *Ae. aegypti* from Brazil compared to *Ae. albopictus* from the United States. However, transmission potential between *Ae. aegypti* and *Ae. albopictus* were similar. These results are consistent with our findings, in that *Ae. aegypti* was more susceptible to ZIKV infection and disseminated infection compared to *Ae. albopictus*. The observed higher leg and body titers in *Ae. aegypti* with disseminated infections is in accordance with observations of higher infection and dissemination rates than *Ae. albopictus,* an indicator of higher permissibility of the former mosquito species for ZIKV. In our study, we provide evidence of variation in midgut and salivary gland barriers in *Ae. aegypti* and *Ae. albopictus* across Florida. 

Higher susceptibility to infection and disseminated infection of Zika virus exhibited in Florida populations of *Ae. aegypti*, together with a preference for human blood [37] and gonotrophic discordance behavior, raises *Ae. aegypti* as the primary vector of Zika virus in Florida, and elsewhere. We can infer from our observations that the higher rates of disseminated infection among Florida *Ae. aegypti* at 14-dpi than *Ae. albopictus* suggests a shorter extrinsic incubation period (EIP) for *Ae. aegypti*, further enhancing risk of Zika transmission. A similar study comparing vector competence measures among Italian populations of *Ae. albopictus* and a long-established colony of *Ae. aegypti* (collected in Reynosa, Mexico, in 1998) and the Asian genotype of Zika virus (similar titers used) observed an EIP_50_ of 7- and 11-days for *Ae. aegypti* and *Ae. albopictus*, respectively [14]. In contrast, the estimated EIP values were longer (16–17 days) for *Ae. albopictus* than *Ae. aegypti* among populations from Florida [38]. A comparison of Australian populations of mosquitoes and a Brazilian ZIKV strain, laboratory colonies of *Ae. aegypti* (Poza Rica, Mexico) and *Ae. albopictus* (Florida) were allowed to ingest ZIKV and held under multiple constant temperatures ranging from 25–35 °C [39]. Vector competence estimates assumed a unimodal pattern with peaks from 28–32 °C, and higher transmission rates occurred on 10-dpi for *Ae. aegypti* and 14-dpi for *Ae. albopictus*. Extrinsic incubation periods were consistently shorter for *Ae. aegypti* than *Ae. albopictus* over a range of temperatures [39]. Discrepancies in results may be attributable, in part, to mosquito and virus strains used and experimental procedures. Overall, our results are consistent with most other studies and extend these patterns of Zika infection to invasive mosquito vectors in Florida.

This project showed relative differences in susceptibility to ZIKV, disseminated infection and transmission rates of *Ae. aegypti* and *Ae. albopictus* vectors, and differences in these rates across Florida. Future studies on vectorial capacity parameters of *Ae. aegypti* and *Ae. albopictus* should be carried out to determine variation among geographic populations to assist with emergence and risk prediction of ZIKV in Florida.

## Figures and Tables

**Table 1:** Geographic origin for Florida populations of *Ae. aegypti* (F4) and *Ae. albopictus* (F4) used in this experiment.

Mosquito Population Collection Site	County	Mosquito Species
Okeechobee, Florida	Okeechobee	*Ae. aegypti*
Okeechobee, Florida	Okeechobee	*Ae. albopictus*
Jacksonville, Florida	Duval	*Ae. albopictus*
Miami, Florida	Miami-Dade	*Ae. aegypti*

**Table 2:** Logistic regression of mosquito species and geographic site on susceptibility to infection (body), disseminated infection (legs), and transmission potential (saliva). The results show the means (probability scale), standard errors, and 95% confidence intervals (lower and upper means).

Mosquito Sample	Species and Geographic Site	Mean (No. Samples)	Std Error of Mean	Lower Mean	Upper Mean
Susceptibility to Infection (Body)	*Ae. aegypti*-Miami	0.2395 (261)	0.0263	0.1918	0.2948
	*Ae. aegypti*-Okeechobee	0.0833 (298)	0.0159	0.0569	0.1204
	*Ae. albopictus*-Okeechobee	0.4606 (341)	0.0269	0.4085	0.5136
	*Ae. albopictus*-Jacksonville	0.5135 (220)	0.0335	0.4479	0.5787
Disseminated Infection (Legs)	*Ae. aegypti*-Miami	0.1324 (134)	0.0290	0.0850	0.2003
	*Ae. aegypti*-Okeechobee	0.1053 (321)	0.0170	0.0761	0.1437
	*Ae. albopictus*-Okeechobee	0.6033 (182)	0.0360	0.5309	0.6714
	*Ae. albopictus*-Jacksonville	0.6789 (107)	0.0447	0.5858	0.7597
Transmission Potential (Saliva)	*Ae. aegypti*-Miami	0.6667 (3)	0.1925	0.2681	0.9161
	*Ae. aegypti*-Okeechobee	0.5246 (59)	0.0633	0.4003	0.6459
	*Ae. albopictus*-Okeechobee	0.7073 (39)	0.0710	0.5522	0.8256
	*Ae. albopictus*-Jacksonville	0.7143 (19)	0.0985	0.4924	0.8656

## Data Availability

The data presented in this study are available on request from the corresponding author.
